# Supplementary material for: Spatial competition constrains resistance to targeted cancer therapy
Source: Nat Commun. 2017 Dec 8;8:1995. doi: 10.1038/s41467-017-01516-1 (PMC5722825; doi:10.1038/s41467-017-01516-1)
Supplement: Supplementary file 1 — Supplementary Information [file 41467_2017_1516_MOESM1_ESM.pdf]

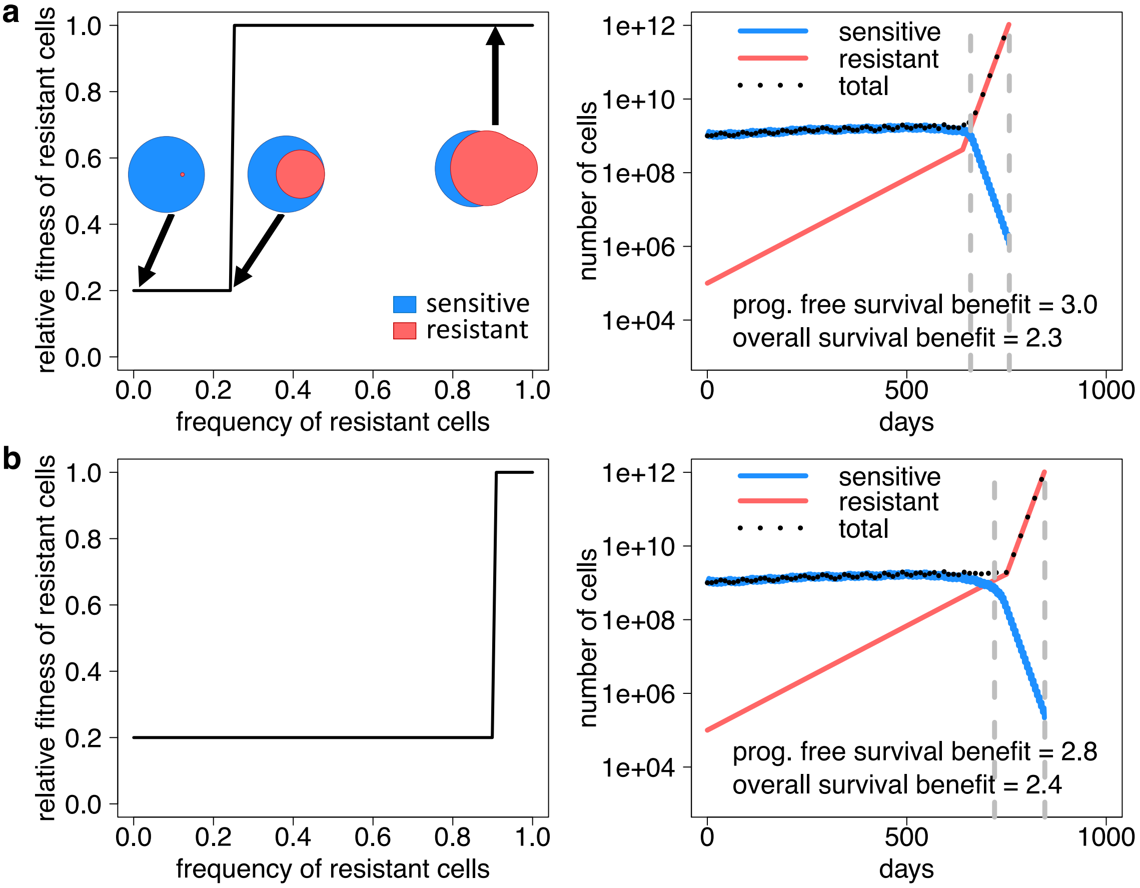


**Supplementary Figure 1. Geometrical analysis of resistant subclone growth within a three-dimensional tumour**

(**a**) In one extreme case, the relationship between the relative fitness and the frequency of resistant cells transitions from its minimum to its maximum value when frequency is 0.25 (left panel, including representative tumour cross sections at various frequencies). Given this relationship, a mathematical model predicts population sizes for sensitive cells (right panel, blue line), resistant cells (red), and all cells (black). The benefit of AT is assessed by measuring the periods of progression-free survival (first vertical dashed line, where the population returns to its initial size) and overall survival (second vertical dashed line, where the population reaches 1012 cells) relative to the corresponding periods under MTD.

(**b**) In the second extreme case, the relationship between the relative fitness and the frequency of resistant cells transitions from its minimum to its maximum value when frequency is 0.9 (left panel), but the relative benefit of AT is similar to that of the previous case.

Parameter values are $\lambda_W = \lambda_R$ = log(2)/10, $IC50_W$ = 1, $IC50_R$ = 100, $\rho_{MTD}$ = 1, θ = 5 days, $N_0 = 10^9$, $k$ = 20, and $c$ = 0.5.

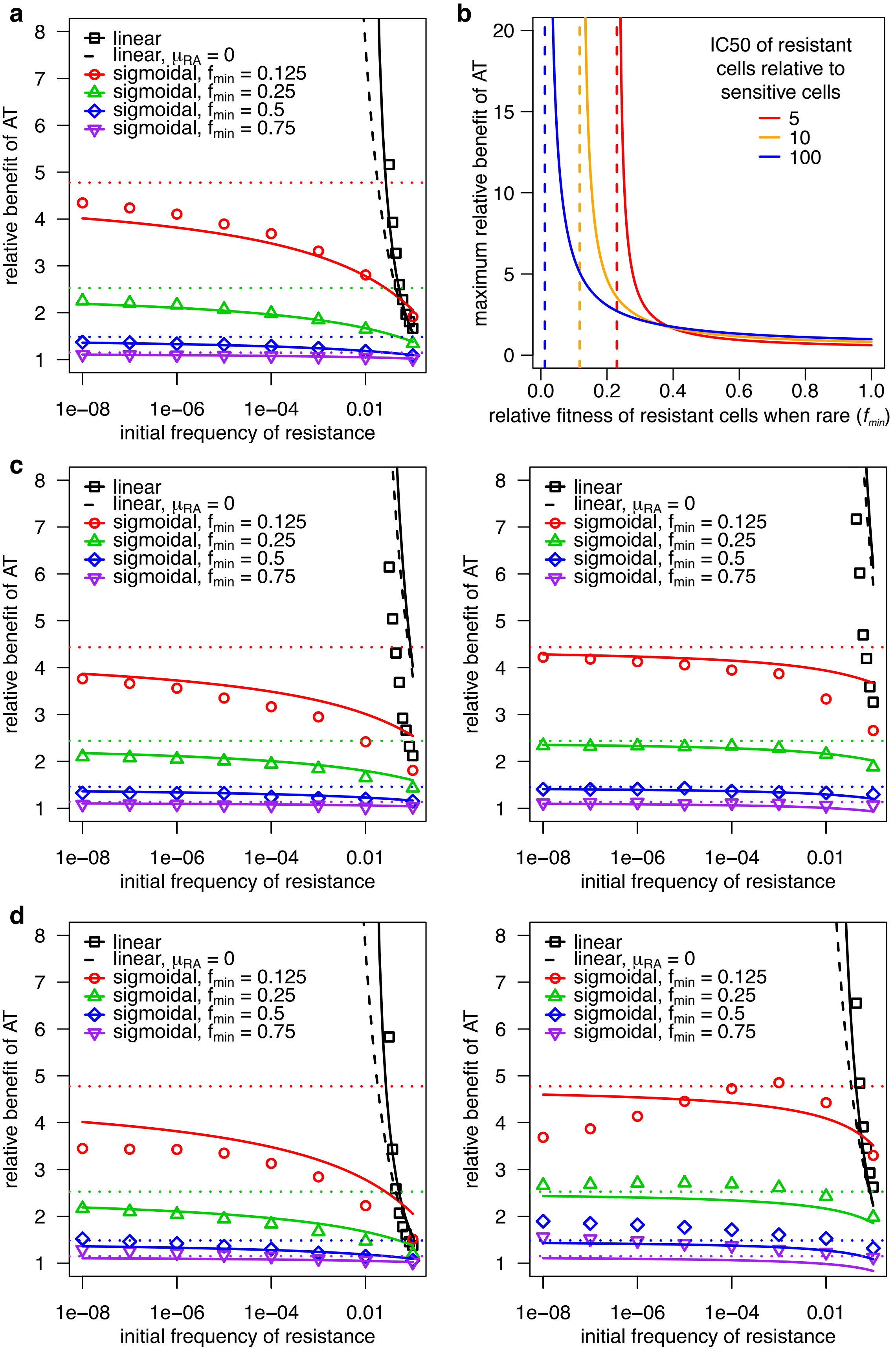
a
linear
linear, μRA = 0
sigmoidal, fmin = 0.125
sigmoidal, fmin = 0.25
sigmoidal, fmin = 0.5
sigmoidal, fmin = 0.75
relative benefit of AT
initial frequency of resistance
1e−08
1e−06
1e−04
0.01
b
IC50 of resistant cells relative to sensitive cells
5
10
100
maximum relative benefit of AT
relative fitness of resistant cells when rare (fmin)
c
d

**Supplementary Figure 2. Mathematical modelling of tumour evolutionary dynamics**
(**a**) Mathematical model results for the overall survival benefit of adaptive therapy (relative to maximum tolerated dose therapy), versus initial frequency of resistant cells. Points represent numerical simulations; curves are approximate analytical solutions; dotted lines are the upper bounds of the approximate analytical solutions. Outcomes are shown for five models assuming different functions $f$ describing the relationship between resistant cell relative fitness and frequency. The first model (black solid curve and square points) assumes a linear function, and assumes that the therapy slightly increases the mortality rate of resistant cells, $\mu_R$. An analytical approximation is also shown for the case $\mu_R = 0$ (dashed black line). Other models assume that $f$ is sigmoidal. In all cases, $\lambda_W = \lambda_R = \log(2)/10$, $\text{IC50}_W = 1$, $\text{IC50}_R = 100$ (except when $\mu_R = 0$), $\rho_{\text{MTD}} = 1$, $\theta = 5$ days, $N_0 = 10^9$, $k = 20$, and $c = 0.5$.
(**b**) Maximum relative survival benefit of adaptive therapy versus $f_{min}$, assuming a sigmoidal function $f$. Curves are shown for different values of the IC50 of CDKi for resistant cells, $\text{IC50}_R$; other parameter values are as in the previous panel. The vertical asymptotes are at $\mu_{RA}/\lambda_R$.
(**c**) Assuming a longer gap between treatment doses, mathematical model results for the progression-free survival benefit (left) and overall survival benefit (right) of adaptive therapy (relative to maximum tolerated dose therapy), versus initial frequency of resistant cells. Parameter values are as in previous panels except $\theta = 30$ days, $\rho_{\text{MTD}} = 63$, $\text{IC50}_R = 1{,}000$.
(**d**) Assuming tumour growth obeys a sigmoidal Gompertz law, mathematical model results for the progression-free survival benefit (left) and overall survival benefit (right) of adaptive therapy (relative to maximum tolerated dose therapy), versus initial frequency of resistant cells. Parameter values are as in the first two panels; the carrying capacity constant in the growth function is $5 \times 10^{12}$.

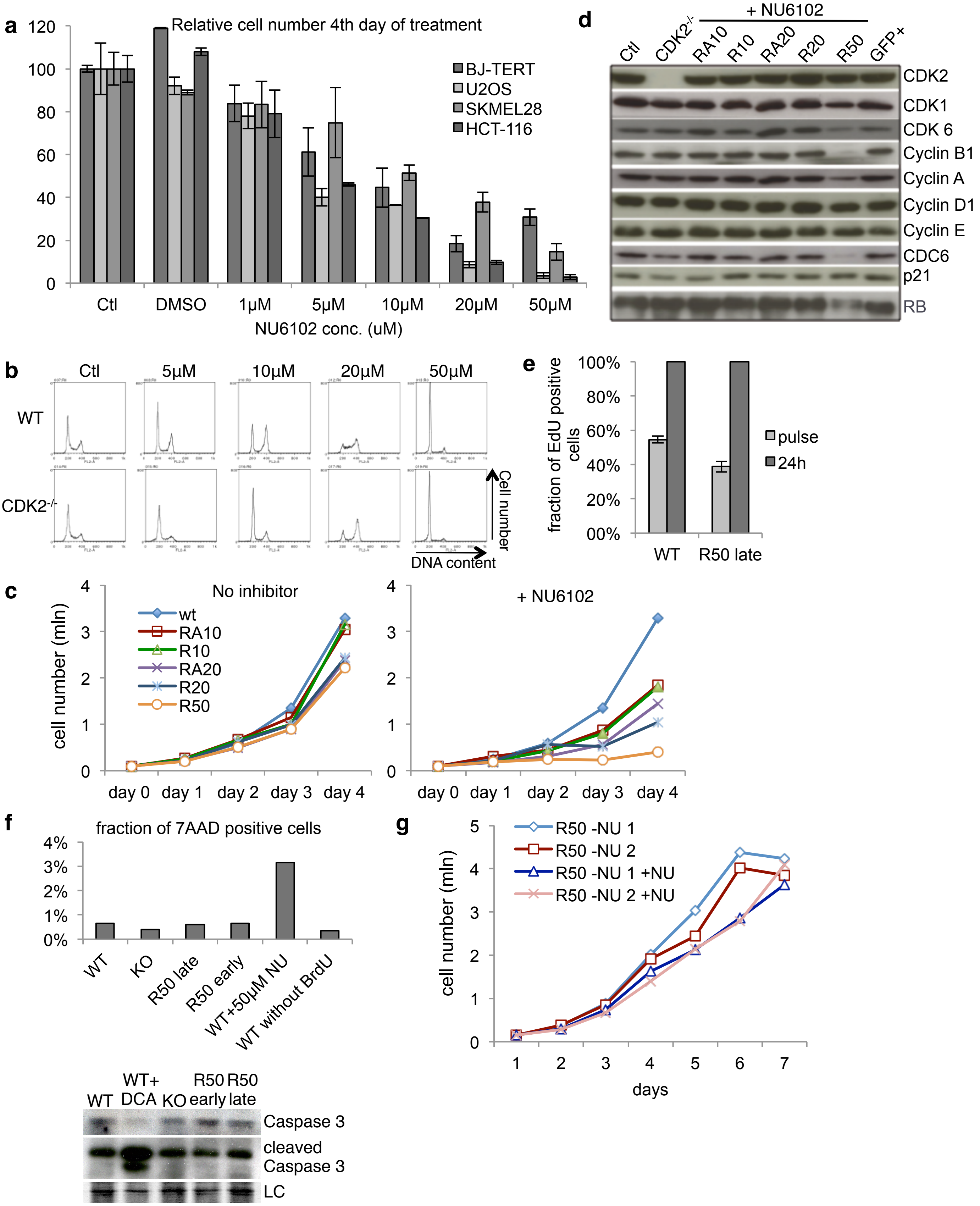
a
Relative cell number 4th day of treatment
BJ-TERT
U2OS
SKMEL28
HCT-116
120
100
80
60
40
20
0
Ctl
DMSO
1µM
5µM
10µM
20µM
50µM
NU6102 conc. (uM)
b
Ctl
5µM
10µM
20µM
50µM
WT
CDK2-/-
Cell number
DNA content
c
No inhibitor
+ NU6102
wt
RA10
R10
RA20
R20
R50
cell number (mln)
day 0
day 1
day 2
day 3
day 4
d
+ NU6102
Ctl
CDK2-/-
RA10
R10
RA20
R20
R50
GFP+
CDK2
CDK1
CDK 6
Cyclin B1
Cyclin A
Cyclin D1
Cyclin E
CDC6
p21
RB
e
fraction of EdU positive cells
100%
80%
60%
40%
20%
00%
pulse
24h
WT
R50 late
f
fraction of 7AAD positive cells
4%
3%
2%
1%
0%
WT
KO
R50 late
R50 early
WT+50µM NU
WT without BrdU
WT
WT+ DCA
KO
R50 early
R50 late
Caspase 3
cleaved Caspase 3
LC
g
R50 -NU 1
R50 -NU 2
R50 -NU 1 +NU
R50 -NU 2 +NU
cell number (mln)
5
4
3
2
1
0
1
2
3
4
5
6
7
days

**Supplementary Figure 3. Characterisation of CDKi-resistant cells**

(**a**) BJ-hTERT, U2OS, SKMEL-28, and HCT-116 cells were grown in control conditions, or in the presence of DMSO or NU6102 at indicated concentrations. Cell number was analysed at day 4 and presented as relative to control sample (mean ± SEM of two independent experiments).

(**b**) Flow cytometry cell cycle profile analysis of WT and CDK2$^{-/-}$ cells grown in control conditions, or treated for 24h with NU6102 at indicated concentrations.

(**c**) WT, RA10, R10, RA20, R20 and early R50 cells were grown in the absence (left) or presence of corresponding concentration of NU6102 (right; WT grown without the drug), and the cell number was analysed for 4 days.

(**d**) Western blot analysis of the indicated proteins in control, CDK2$^{-/-}$, RA10, R10, RA20, R20, R50 and WT-GFP+ cells (resistant cells grown in the presence of corresponding concentrations of NU6102).

(**e**) Percentage of EdU positive WT and R50 cells, either pulsed for 15min or incubated with EdU for 24h (mean ± SD of two independent experiments).

(**f**) Analysis of cell viability (top, 7AAD staining) and apoptosis (bottom, western blot staining for cleaved Caspase 3) in WT, CDK2$^{-/-}$ (KO), early and late R50. 24h treatment with 50μM NU6102 of WT cells was used as control for cell death; DCA (deoxycholic acid) treatment for apoptosis.

(**g**) R50 cells were maintained for 2 (-NU1) or 6 (-NU2) months without NU6102. Subsequently, their proliferation was evaluated in the presence (+NU) or absence of the drug.

a

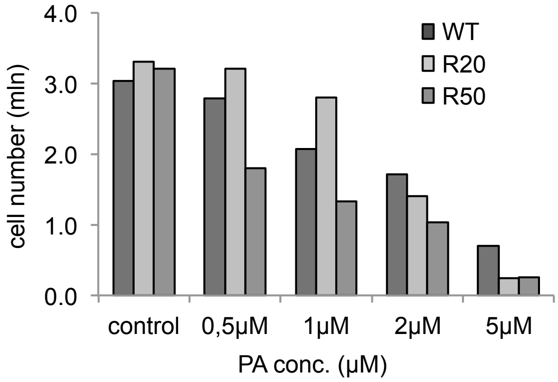


b

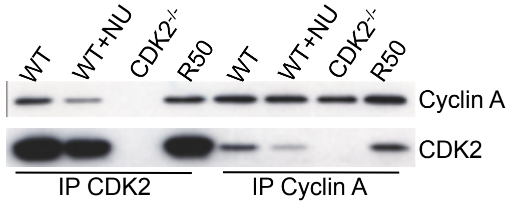


c

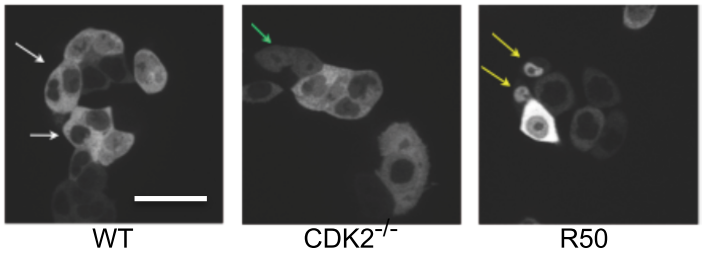


d

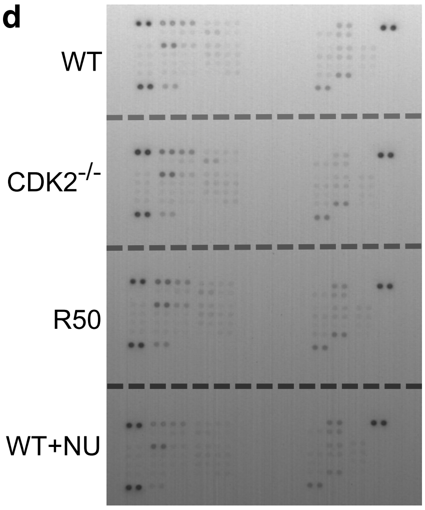


e

| R50 vs WT | R50vs WT+NU |
|---|---|
| Pathways in cancer (p=4.9E-07) | Cell cycle (p=5.3E-10) |
| Endocytosis (p=2.7E-06) | RNA transport (p=1.9E-08) |
| Focal adhesion (p=3.3E-05) | Protein processing in endoplasmic reticulum (p=4.3E-07) |
| Glycerolipid metabolism (p=5E-05) | Phagosome (p=4.8E-07) |
| Purine metabolism (p=5E-05) | Pathways in cancer (p=5.4E-07) |
| Protein processing in endoplasmic reticulum (p=5.7E-05) | Endocytosis (p=1.7E-06) |

f

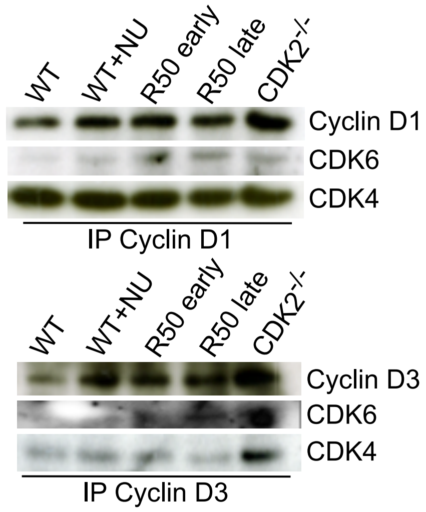


g

| | CDK2 /cycA2 | CDK4 /cycD1 | CDK4 /cycD3 | CDK6 /cycD1 | CDK6 /cycD3 |
|---|---|---|---|---|---|
| **Vmax** | 251000 ± 6500 | 238000 ± 16000 | 149000 ± 46000 | 281000 ± 6000 | 255000 ± 60000 |
| **Km (ATP) / µM** | 9 ± 1.1 | 54 ± 10 | 338 ± 151 | 15 ± 1.3 | 154 ± 66 |
| **Ki (NU6102) / nM** | 5.7 ± 0.4 | 35 ± 3 | 285 ± 102 | 23 ± 4 | 14 ± 8 |

**Supplementary Figure 4. Mechanisms of cell cycle rewiring in CDKi-resistant cells**
(**a**) WT, R20 and R50 cells were grown for 24h in the presence of the indicated concentrations of purvalanol A (PA) and their number was analysed. Representative of two experiments.
(**b**) CDK2 and cyclin A were immunoprecipitated from WT, WT cells grown with 20μM NU6102 for 24h, CDK2$^{-/-}$ and R50 cells, and CDK2/cyclin A complexes were analysed by western blotting.
(**c**) Immunofluorescence images of WT, CDK2$^{-/-}$ and R50 cells expressing DHB-Venus CDK2 activity sensor (white arrow, cytoplasmic localisation; green arrow, both cytoplasmic and nuclear; yellow arrow, nuclear). Bar, 50μm.
(**d**) Profiling of signalling pathways in WT, WT cells grown with 20μM NU6102 for 24h, CDK2$^{-/-}$ and R50 cells using Human Phospho-Kinase Array membranes (R&D systems) in sandwich immunoassays with chemiluminescent detection. The results are representative of two independent experiments.
(**e**) KEGG pathway analysis of transcriptome data presented in Figure 2c. T-test analysis was performed on genes altered between R50 and WT, and R50 and WT cells treated for 24h with 20μM NU6102.
(**f**) Cyclin D1 and cyclin D3 were immunoprecipitated from WT, WT cells treated with 20μM NU6102 for 24h, CDK2$^{-/-}$, and early and late R50 cells, and beads were blotted for associated cyclins CDK4 and CDK6.
(**g**) Purified recombinant kinases were used in *in vitro* assays with variable ATP concentrations to determine the Michaelis constant $K_m$, and with constant ATP but with variable NU6102 concentrations to assess the inhibitor constant $K_i$. Values represent mean ± SEM of two replicates, from one representative assay.

Supplementary Figure 5.

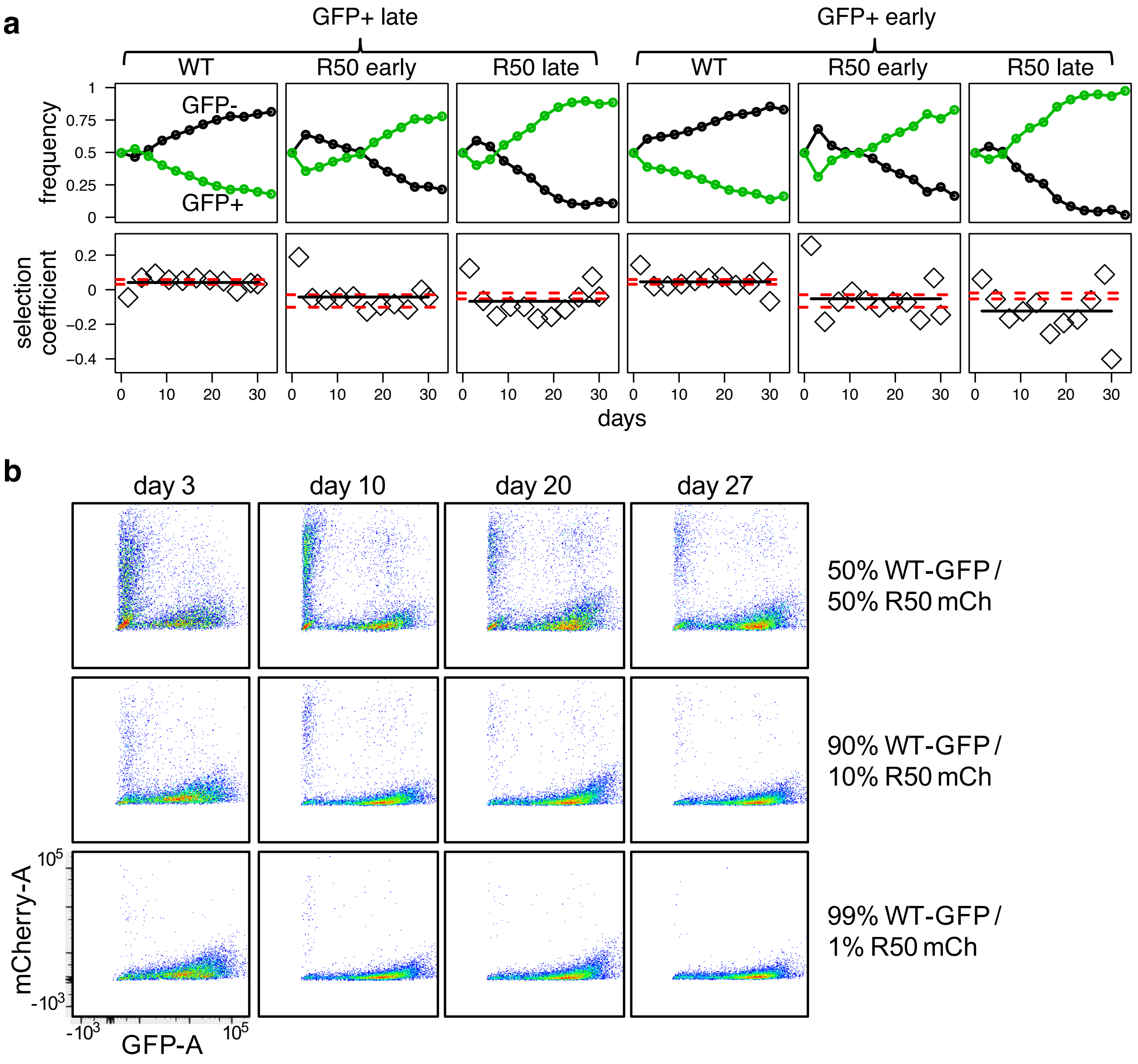


**Supplementary Figure 5. Resistant cells are outcompeted by sensitive cells in the absence of selection**
(**a**) Frequency dynamics (top row) and selection coefficients for competition assays, compared to predictions from growth rates (bottom row). In the bottom row, each point corresponds to a selection coefficient calculated from a competition assay (i.e. a period between consecutive points in the top row) and solid lines are means. Red dashed lines indicate predictions based on the growth rates of each cell type in isolation (Supplementary materials). A single prediction is shown whenever growth curves were measured at the same time as competitions were conducted; otherwise pairs of lines show maximum and minimum predictions based on non-contemporaneous growth curves. If selection coefficients do not coincide with the prediction then an ecological interaction between the two cell types is suggested. GFP+ CDKi-sensitive cells from two different time points (GFP+ late and GFP+ early) were competed against GFP- CDKi-sensitive cells (WT), GFP- R50 early cells and GFP- R50 late cells. Representative of 3 independent experiments.
(**b**) Flow cytometry analysis of the percentage of GFP+ and mCh+ cells, seeded initially at the indicated ratios, from monolayer competition experiment presented in Fig. 3f.

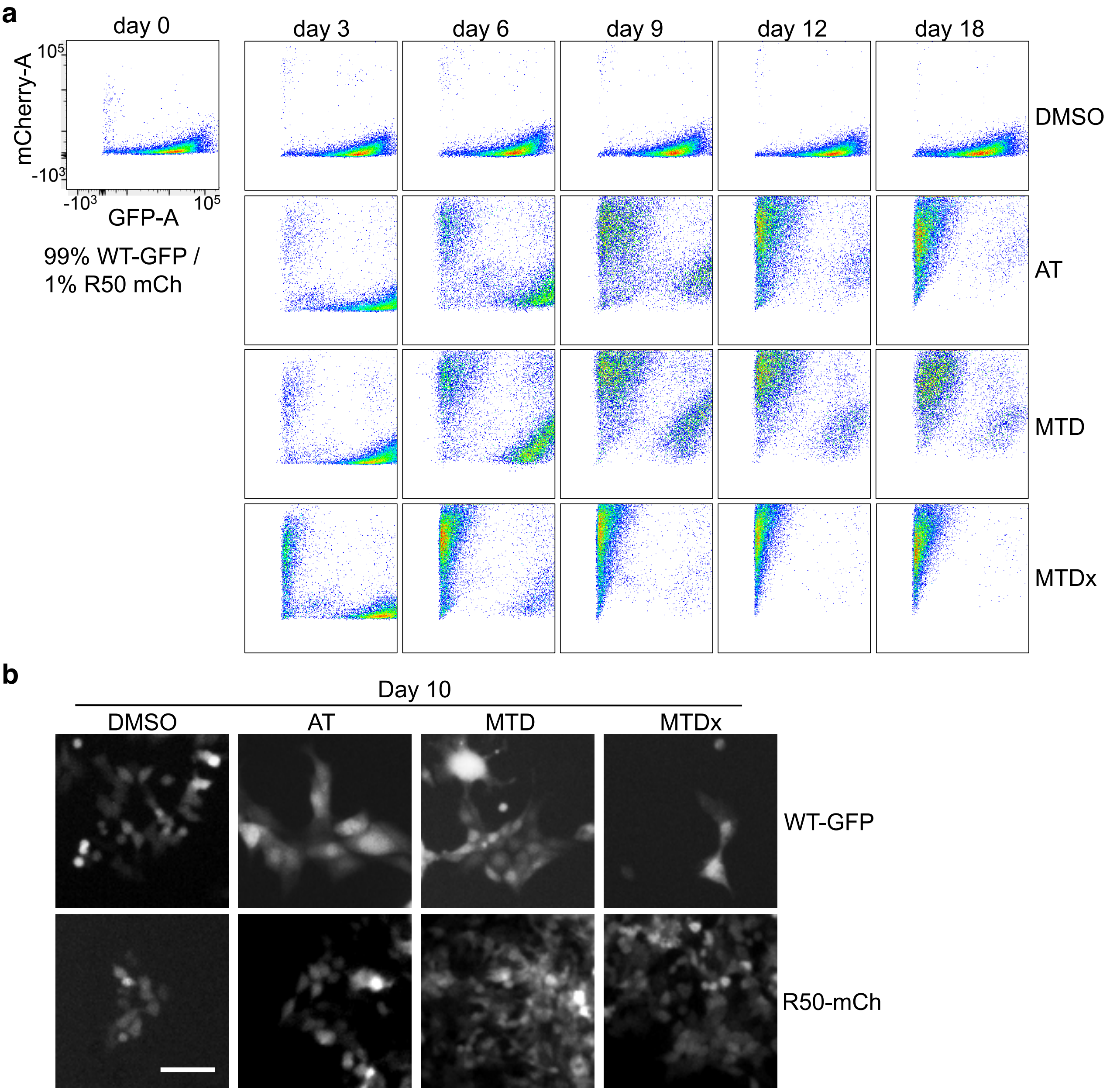


**Supplementary Figure 6. AT does not outperform MTD in limiting tumour growth in 2D**

(**a**) Flow cytometry analysis of WT-GFP+ and R50-mCh+ cell populations from experiment presented in Figure 4, at indicated time points.

(**b**) Immunofluorescent images of WT-GFP+ and R50-mCh+ cells from the experiment in (**a**) at day 10 (bar, 100µm).

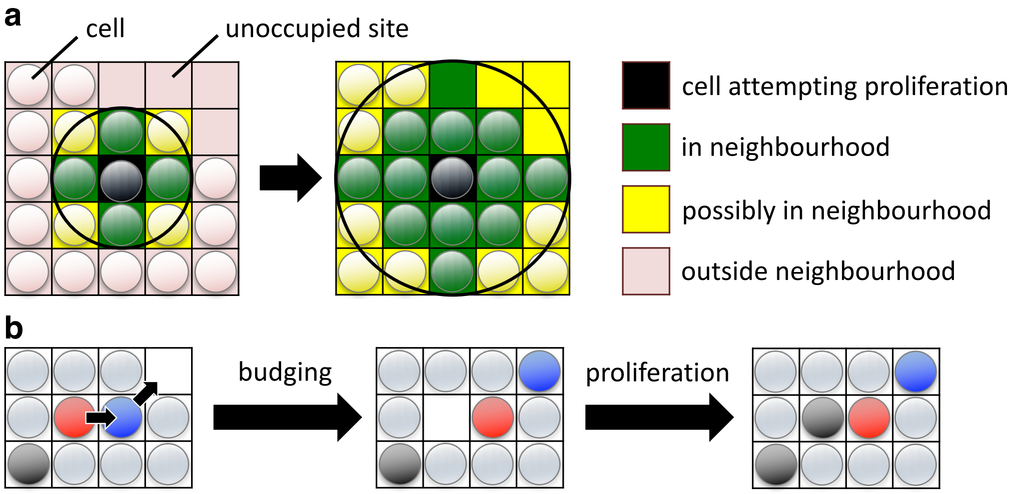


**c**

| Dose | Proliferation rate, $P$ | | Death rate, $M$ | |
|---|---|---|---|---|
| | Sensitive | Resistant | Sensitive | Resistant |
| 0 | 1 | 0.9 | 0 | 0 |
| 5 | 0.75 | 0.9 | 0 | 0 |
| 10 | 0.50 | 0.9 | 0 | 0 |
| 20 | 0.002 | 0.9 | 0.003 | 0 |
| 50 | 0 | 0.9 | 0.55 | 0 |

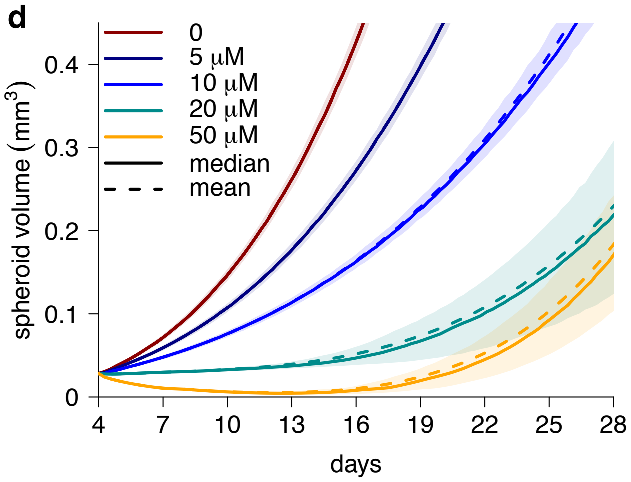


**Supplementary Figure 7. Assumptions of the spatial computational model**
(**a**) In the spatial computational model, a cell can proliferate only if it has nearby unoccupied space. First, the algorithm looks for an unoccupied site (that is, a site containing either medium or a dead cell) within 1.5 cell diameters of the centre of the focus (black) cell by checking sites in random order. Sites entirely within the search disc (green) are always checked; sites partially within the disc (yellow) are checked with probability proportional to their overlap. If no unoccupied site is found then the search radius is expanded by one cell diameter. The process continues until the radius reaches a limit *d*.
(**b**) If the first unoccupied site to be found is not adjacent to the focus cell then the cells lying on the straight line between the focus cell and the unoccupied site are shifted by one site to create a space adjacent to the focus cell. This adjacent space is filled by the new daughter cell.
(**c**) Values of proliferation and death rates of cells at maximal oxygen concentration in the computational model, versus CDK inhibitor dose.
(**d**) Tumour spheroid growth curves for different treatment regimens in the computational model when *d* = 20. Medians (solid curves), means (dashed curves) and interquartile ranges (shaded) are shown for 1,000 stochastic simulations.

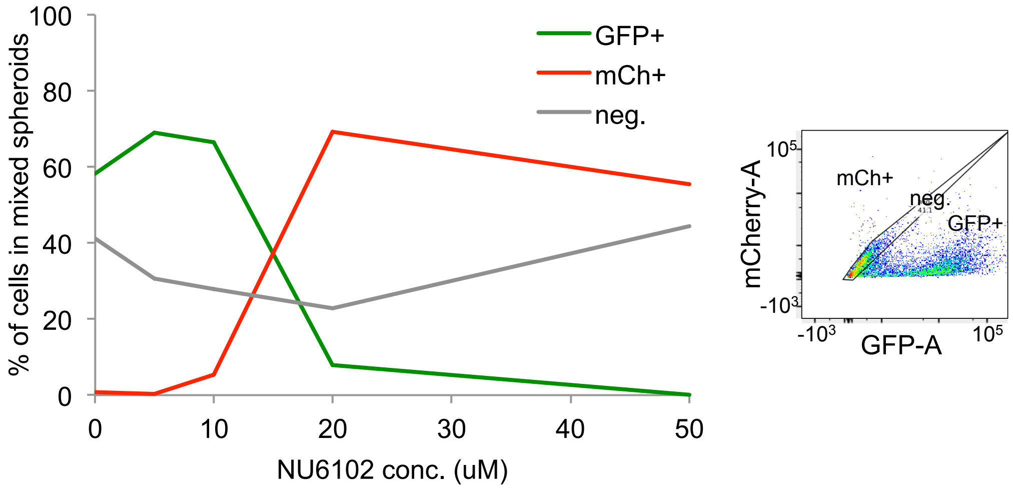


**Supplementary Figure 8. Effective restraint of tumour growth in spheroids by low drug concentrations**

Percentage of GFP+, mCh+ and negative cells within mixed spheroids at day 28, grown at indicated concentrations of NU6102, measured by flow cytometry analysis (as in Fig. 6b). Right, representative flow cytometry histogram illustrating the different cell populations.

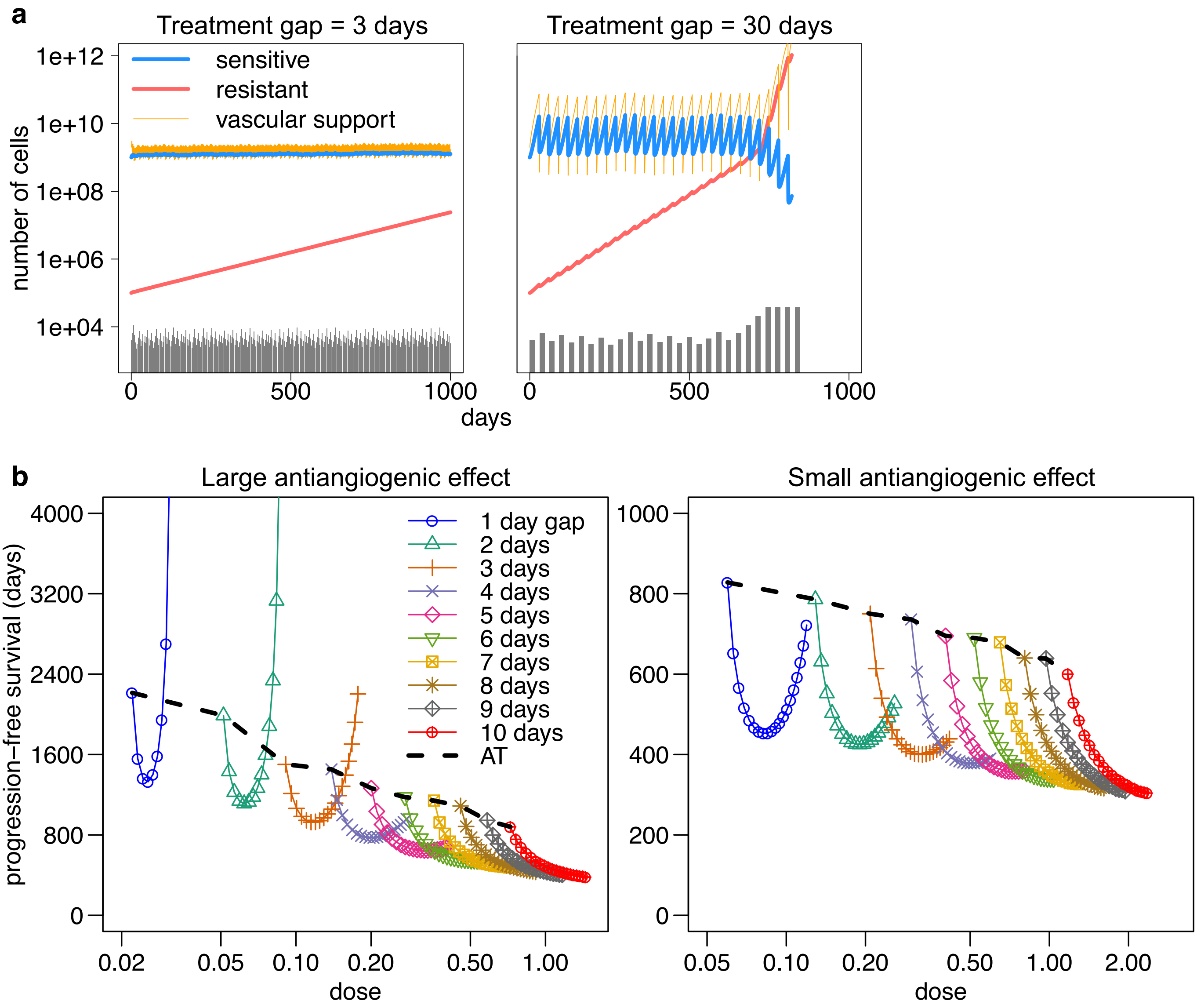


**Supplementary Figure 9.**
(**a**) Example results of an expanded mathematical model in which tumour growth is limited by a carrying capacity linked to vascular support, which can change in response to tumour size and the treatment's antiangiogenic effect. The resistant cell population expands more slowly when the gap between AT doses is short (left panel) than when it is longer (right panel) because the tumour's vascular support (thin orange line) temporarily recovers in the absence of treatment. Grey bars show how the AT dose changes over time. Parameter values are $b$ = 0.5, $d = 10^{-9}$, $K_0 = 2\times10^9$, $\lambda_W = \lambda_R$ = log(2)/10, $IC50_W$ = 1, $IC50_R$ = 100, $IC50_K$ = 0.1, $N_0$ = 109, $R_0 = 10^5$, $k$ = 20, and $c$ = 0.5.
(**b**) In the expanded model, progression-free survival time varies with treatment schedule. Survival times are shown for different combinations of treatment gaps and doses. For each value of the treatment gap, the range of doses varies from the AT dose (which is the minimum required to prevent tumour growth) up to double the AT dose (assuming that higher doses would be intolerable). Higher dose metronomic therapy is superior to AT if the treatment has a relatively strong antiangiogenic effect and the treatment gap is small (left panel with treatment gap less than 4 days); otherwise, AT is optimal (right panel, and left panel with treatment gap of 4 days or more). Dashed lines show how survival time varies with treatment gap for AT only. Parameter values are as above except $IC50_K$ = 0.5 in the right panel.

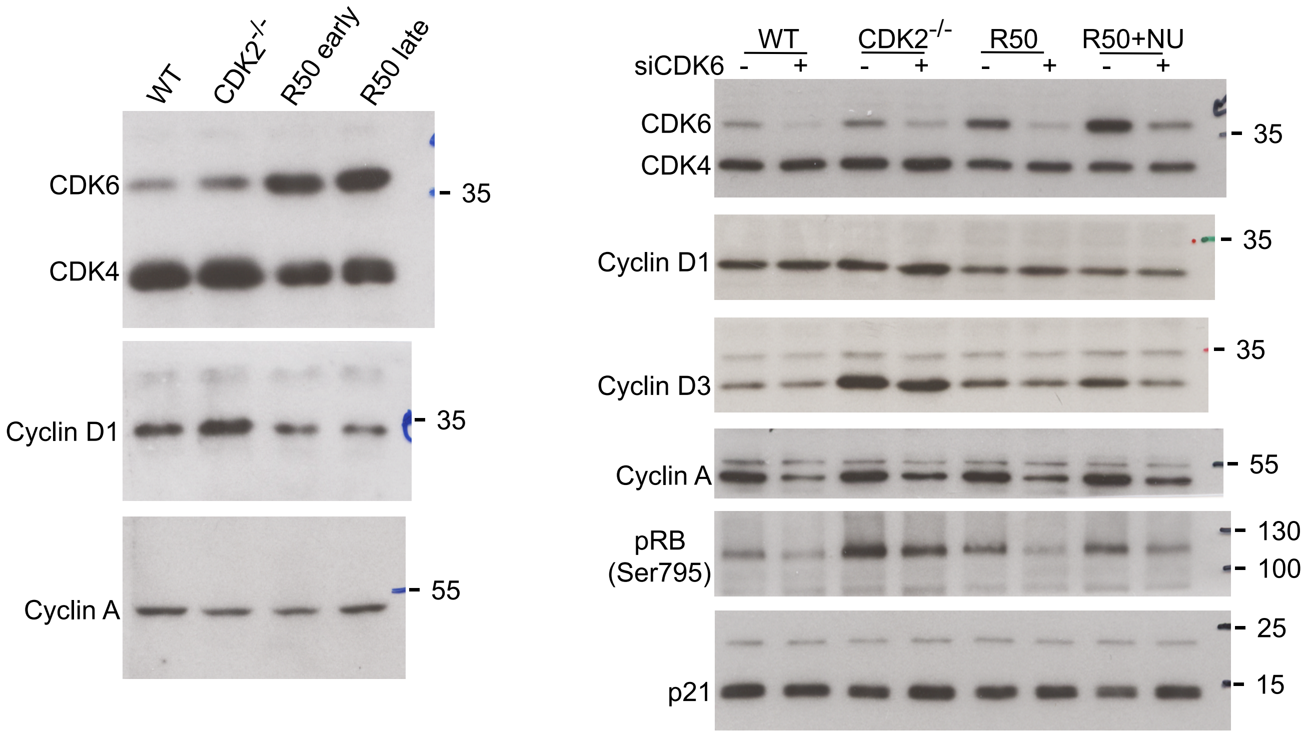


**Supplementary Figure 10.**
Uncropped Western blots from Figure 2.

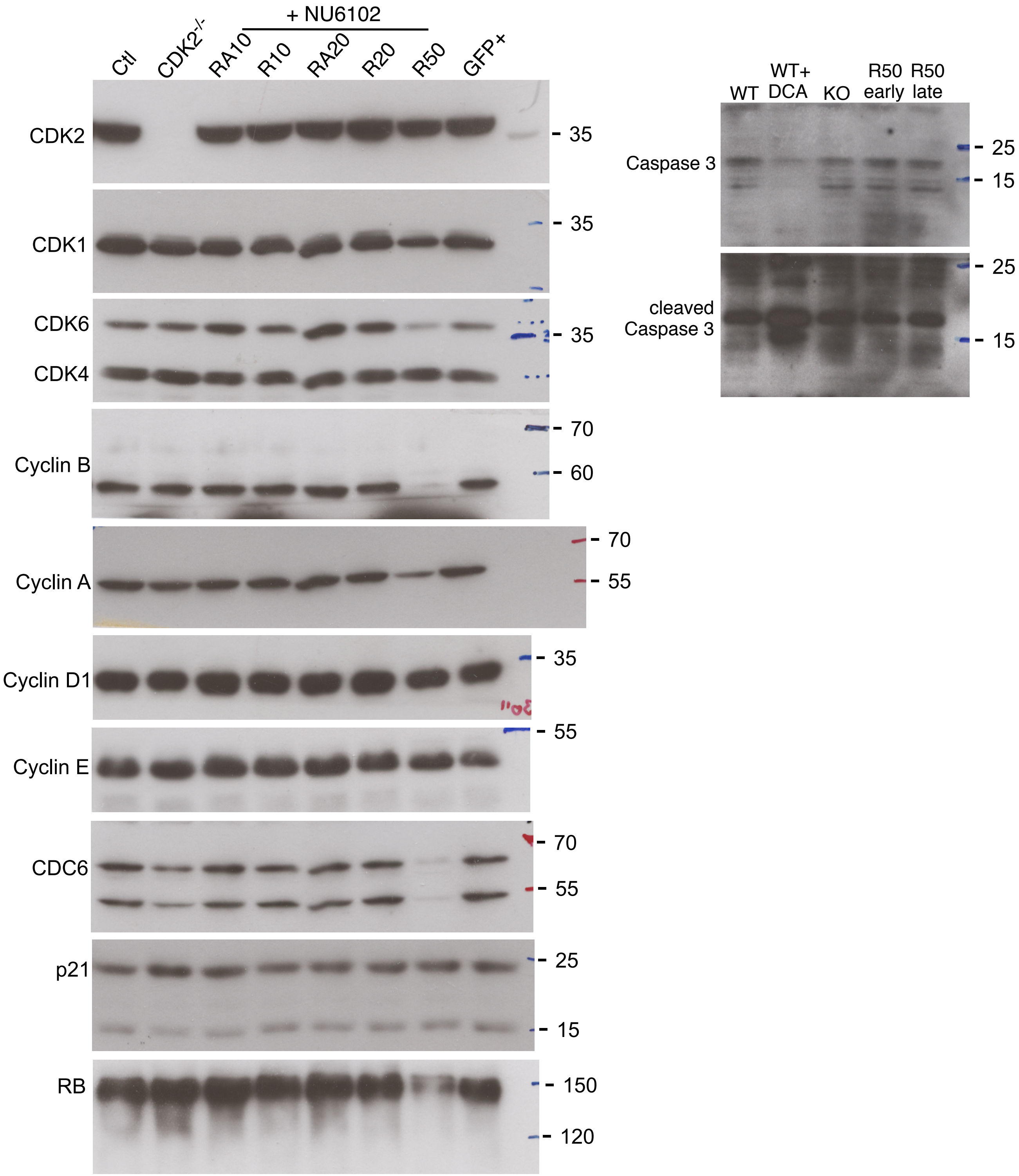


**Supplementary Figure 11.**
Uncropped Western blots from Supplementary Figure 3.

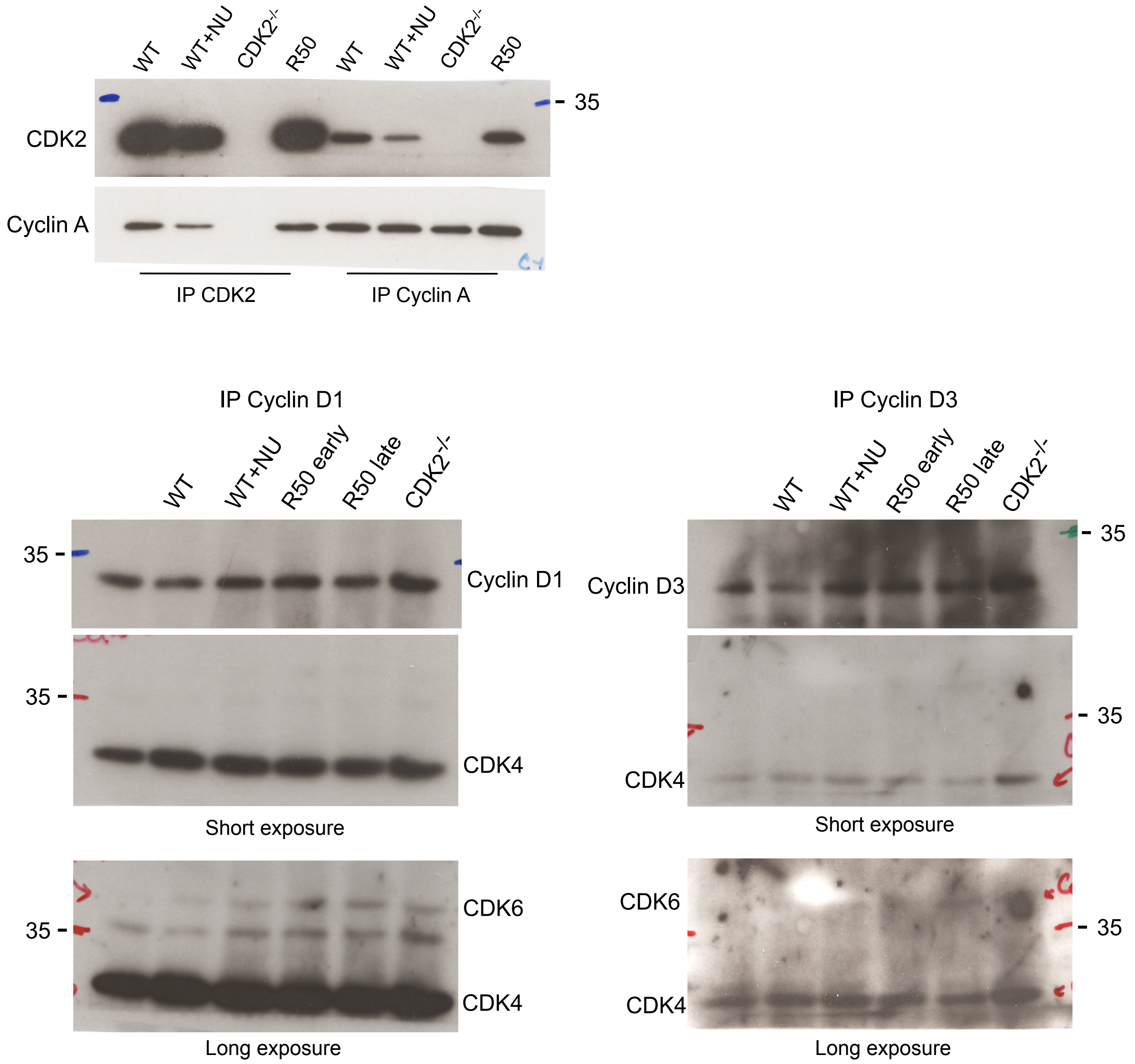


**Supplementary Figure 12.**
Uncropped Western blots from Supplementary Figure 4.

# Supplementary methods

## Non-spatial mathematical model

### Frequency dependent fitness of resistant cells

Adaptive therapy (AT) depends on the axiom that chemosensitive cells suppress the proliferation of chemoresistant cells. It follows that resistant cells must be less fit when rare than when abundant, and, in the absence of evidence to the contrary, it is reasonable to assume that fitness increases monotonically between these two extremes. The problem then is to determine the shape of this monotonic relationship. Since little is known in general about the multitudinous potential interactions between tumour subclones, our solution must be somewhat speculative, yet a few basic considerations suggest that certain functional forms are more plausible than others. We further narrow the field of candidates by focussing on obtaining upper bounds for the benefit of AT versus maximum tolerated dose (MTD) therapy.

We begin by assuming that, on average, a resistant subclone will be less fit when it is surrounded by chemosensitive cells than when it borders healthy tissue or lumen. We further assume that the first resistant subclone to reach the tumour periphery is equally likely to have arisen at any point in the tumour. This latter assumption will be unrealistic if resistance mutations occur more often near the tumour periphery (e.g. due to higher cell turnover) or if multiple subclones are present at different locations. Therefore this assumption is likely to overestimate the period for which resistance is suppressed, which makes it appropriate for determining the upper bound of AT effectiveness.

Assuming symmetric growth, we calculate the expected volume of the resistant subclone at the time it reaches the tumour periphery, relative to the entire tumour volume, by evaluating

$$\int_0^1 x^3 dx = 1/4, \tag{1}$$

where $x$ is the resistant subclone radius relative to the tumour radius. This means that, in the best case scenario, the resistant subclone is expected to be surrounded by chemosensitive cells until its frequency reaches 0.25. As we are interested in the upper bound of AT benefit, we assume that the relative fitness of resistant cells remains at its minimum value during this initial growth period. Near the other extreme, when

resistant cells comprise more than 90% of the tumour, suppression by the remaining chemosensitive cells is likely to be negligible, and we can reasonably approximate that the relative fitness of resistant cells achieves its maximum value.

Fitness during the intermediate period, when frequency is between 0.25 and 0.9, is uncertain. We therefore consider the two extreme cases, in which the relative fitness of resistant cells is a step function that transitions from its minimum to its maximum value when frequency is either 0.25 or 0.9 (left panels of Supplementary Fig. 1A and 1B, respectively). For plausible parameter values, the predictions of our mathematical model are highly similar for these two extreme cases (right panels of Supplementary Fig. 1A and 1B). This is because the 0.25-0.9 frequency range covers only a small part of the growth curves, and shifting the transition point from 0.25 to 0.9 increases survival time under MTD as well as AT, so that the ratio of the survival times remains similar. Hence our results are insensitive to the exact shape of the relative fitness curve at intermediate frequencies. Indeed, the analytical approximations that we derive below are equally consistent with either of the extreme-case step functions.

For our numerical simulations we assume a smooth sigmoidal relative fitness function (SRF) that approximates the average of all potential solutions. We parametrize the function as a logistic curve:

$$f(R, W) = f_{min} + \frac{1 - f_{min}}{1 + \exp\left(-k\left(\frac{R}{R+W} - c\right)\right)}, \tag{2}$$

where $W$ and $R$ are the chemosensitive and resistant populations, respectively; $f_{min}$ is the minimum relative fitness; and $k$ and $c$ determine the shape of the curve. In this case, the effectiveness of AT strongly depends on parameter $f_{min}$.

For comparison with previous research, we also obtain results for a linear relative fitness function (LRF). A linear relationship between the relative fitness and the frequency of the resistant subpopulation is

$$f(R, W) = \frac{R}{R+W}. \tag{3}$$

This may be considered a relatively favourable scenario for adaptive therapy because it means that resistant cells are relatively very unfit when rare. According to the LRF, the outcome of AT depends strongly on the initial frequency of resistant cells.

The model is then defined as

$$\frac{dW}{dt} = \lambda_W W, \quad \frac{dR}{dt} = \lambda_R f(R, W) R, \tag{4}$$

where $W$ and $R$ are the chemosensitive and resistant populations, respectively; $\lambda_W$ and $\lambda_R$ are the maximum growth rates; and $f$ is a frequency-dependent relative fitness function (either SRF or LRF).

In the mathematical analysis that follows, we assume exponential growth and we exploit the fact that the dynamics under both MTD and AT can be broadly partitioned into two phases. During period 1, the resistant subpopulation is at low frequency, and it therefore grows at a relatively slow, frequency-dependent rate. In period 2, the resistant subpopulation is at high frequency and grows approximately exponentially.

## Average death rates

During MTD at dose $\rho_{MTD}$, the time-averaged death rates of sensitive and resistant cells are respectively

$$\mu_W = \frac{1}{\theta}\log\left(1+\frac{\rho_{MTD}}{IC50_W}\right), \quad \mu_{RM} = \frac{1}{\theta}\log\left(1+\frac{\rho_{MTD}}{IC50_R}\right), \tag{5}$$

where $\theta$ is the period between treatments. The AT dose required to maintain the sensitive population at a constant mean size is

$$\rho_{AT} = (e^{\theta\lambda_W}-1)IC50_W, \tag{6}$$

and so the time-averaged death rate of resistant cells during AT is

$$\mu_{RA} = \frac{1}{\theta}\log\left(1+\frac{\rho_{AT}}{IC50_R}\right) = \frac{1}{\theta}\log\left(1+(e^{\theta\lambda_W}-1)\frac{IC50_W}{IC50_R}\right). \tag{7}$$

## Period 1 (MTD)

During period 1 under MTD, the sensitive population declines exponentially as

$$\frac{dW}{dt} = (\lambda_W - \mu_W)W. \tag{8}$$

This equation has solution

$$W = W_0 e^{(\lambda_W-\mu_W)t}, \tag{9}$$

where $W_0$ is the initial resistant subpopulation.

In the LRF model, the resistant subpopulation grows relatively little during this phase, and we can approximate it as constant.

For the SRF model, if $R_0 \ll W_0$ then $R/W$ will be relatively small for most of the duration of treatment, and so

$$f(R,W) \approx f_{min}. \tag{10}$$

We then have

$$\frac{dR}{dt} \approx (\lambda_R f_{min} - \mu_{RM})R, \tag{11}$$

which has solution

$$R \approx R_0 e^{(\lambda_R f_{min}-\mu_{RM})t}. \tag{12}$$

## Period 1 (AT)

During period 1 under AT, the sensitive subpopulation remains approximately constant. For the LRF model, we can then approximate

$$\frac{dR}{dt} \approx \frac{\lambda_R R^2}{N_0} - \mu_{RA} R, \tag{13}$$

where $N_0$ is the initial total population. This has solution

$$R = \begin{cases} \dfrac{\mu_{RA} N_0 R_0}{\lambda_R R_0 - (\lambda_R R_0 - \mu_{RA} N_0) e^{\mu_{RA} t}} & \text{if } \mu_{RA} > 0 \\ \dfrac{N_0 R_0}{N_0 - \lambda_R R_0 t} & \text{if } \mu_{RA} = 0, \end{cases} \tag{14}$$

where $R_0$ is the initial resistant population.

During period 1 in the SRF model, similarly to the MTD case we have

$$\frac{dR}{dt} \approx (\lambda_R f_{min} - \mu_{RA}) R, \tag{15}$$

and so

$$R \approx R_0 e^{(\lambda_R f_{min} - \mu_{RA}) t}. \tag{16}$$

## Period 2

During period 2 in each model and under either therapy, the resistant subpopulation grows approximately exponentially as

$$\frac{dR}{dt} \approx (\lambda_R - \mu_{RM}) R, \tag{17}$$

which has solution

$$R \approx R_1 e^{(\lambda_R - \mu_{RM}) t}, \tag{18}$$

where $R_1$ is the resistant subpopulation size at the end of period 1.

## Predicted benefits of AT

The predicted survival benefit of AT, relative to MTD, is

$$\frac{T_{AT1} + T_{AT2}}{T_{MTD1} + T_{MTD2}}, \tag{19}$$

where $T_{AT1}$ and $T_{AT2}$ are the durations of periods 1 and 2 (respectively) under AT, and $T_{MTD1}$ and $T_{MTD2}$ are the durations of periods 1 and 2 (respectively) under MTD.

Under MTD, the transition between periods occurs when the sizes of the sensitive and resistant subpopulations are equal. Under AT, the transition happens when the resistant subpopulation size reaches $cN_0$, where $c$ is the inflection point of the fitness function (i.e. the frequency at which resistant cell fitness is halfway between its minimum and maximum values). Period 2 finishes when the total population reaches the endpoint size. When measuring progression-free survival, the endpoint size is $N_0$; for overall survival, it is some larger population size that we denote $M$.

It follows that when measuring overall survival in the LRF model,

$$
\begin{aligned}
T_{MTD1} &= \frac{1}{\lambda_W - \mu_W} \log\left(\frac{R_0}{W_0}\right), & T_{MTD2} &= \frac{1}{\lambda_R - \mu_{RM}} \log\left(\frac{M}{R_0}\right), \\
T_{AT1} &= \begin{cases} \frac{1}{\mu_{RA}} \log\left(\frac{R_0(\lambda_R - \mu_{RA}/c)}{\lambda_R R_0 - \mu_{RA} N_0}\right) & \text{if } \mu_{RA} > 0 \\ \frac{1}{\lambda_R}\left(\frac{N_0}{R_0} - \frac{1}{c}\right) & \text{if } \mu_{RA} = 0, \end{cases} & T_{AT2} &= \frac{1}{\lambda_R - \mu_{RM}} \log\left(\frac{M}{cN_0}\right).
\end{aligned} \tag{20}
$$

If $\mu_{RA} > 0$ then the predicted overall survival benefit of AT, relative to MTD, for the LRF model is

$$
\begin{aligned}
&\frac{\frac{1}{\mu_{RA}} \log\left(\frac{R_0(\lambda_R - \mu_{RA}/c)}{\lambda_R R_0 - \mu_{RA} N_0}\right) + \frac{1}{\lambda_R - \mu_{RM}} \log\left(\frac{M}{cN_0}\right)}{\frac{1}{\lambda_W - \mu_W} \log\left(\frac{R_0}{W_0}\right) + \frac{1}{\lambda_R - \mu_{RM}} \log\left(\frac{M}{R_0}\right)} \\
&= \frac{\frac{\lambda_R - \mu_{RM}}{\mu_{RA}} \log\left(\frac{R_0(\lambda_R - \mu_{RA}/c)}{\lambda_R R_0 - \mu_{RA} N_0}\right) + \log\left(\frac{M}{cN_0}\right)}{\frac{\lambda_R - \mu_{RM}}{\lambda_W - \mu_W} \log\left(\frac{R_0}{W_0}\right) + \log\left(\frac{M}{R_0}\right)},
\end{aligned} \tag{21}
$$

and if $\mu_{RA} = 0$ then it is

$$
\frac{\frac{\lambda_R - \mu_{RM}}{\lambda_R}\left(\frac{N_0}{R_0} - \frac{1}{c}\right) + \log\left(\frac{M}{cN_0}\right)}{\frac{\lambda_R - \mu_{RM}}{\lambda_W - \mu_W} \log\left(\frac{R_0}{W_0}\right) + \log\left(\frac{M}{R_0}\right)}. \tag{22}
$$

When measuring progression-free survival in the LRF model, $T_{MTD1}$ and $T_{AT1}$ are as in the case of overall survival but

$$
T_{MTD2} = \frac{1}{\lambda_R - \mu_{RM}} \log\left(\frac{N_0}{R_0}\right), \quad T_{AT2} = 0. \tag{23}
$$

The predicted progression-free survival benefit of AT, relative to MTD, for the LRF

model is therefore

$$\begin{cases} \dfrac{\frac{\lambda_R-\mu_{RM}}{\mu_{RA}}\log\left(\frac{R_0(\lambda_R-\mu_{RA}/c)}{\lambda_R R_0-\mu_{RA}N_0}\right)}{\frac{\lambda_R-\mu_{RM}}{\lambda_W-\mu_W}\log\left(\frac{R_0}{W_0}\right)+\log\left(\frac{N_0}{R_0}\right)} & \text{if } \mu_{RA}=0, \\ \dfrac{\frac{\lambda_R-\mu_{RM}}{\lambda_R}\left(\frac{N_0}{R_0}-\frac{1}{c}\right)}{\frac{\lambda_R-\mu_{RM}}{\lambda_W-\mu_W}\log\left(\frac{R_0}{W_0}\right)+\log\left(\frac{N_0}{R_0}\right)} & \text{otherwise.} \end{cases} \tag{24}$$

In the SRF model, when measuring overall survival,

$$\begin{aligned} T_{MTD1} &= \frac{1}{\lambda_W-\mu_W-(\lambda_R f_{min}-\mu_{RM})}\log\left(\frac{R_0}{W_0}\right), \\ T_{MTD2} &= \frac{1}{\lambda_R-\mu_{RM}}\left[\log\left(\frac{M}{R_0}\right)-\frac{\lambda_R f_{min}-\mu_{RM}}{\lambda_W-\mu_W-(\lambda_R f_{min}-\mu_{RM})}\log\left(\frac{R_0}{W_0}\right)\right], \\ T_{AT1} &= \frac{1}{\lambda_R f_{min}-\mu_{RA}}\log\left(\frac{cN_0}{R_0}\right), \\ T_{AT2} &= \frac{1}{\lambda_R-\mu_{RM}}\log\left(\frac{M}{cN_0}\right). \end{aligned} \tag{25}$$

Therefore the predicted overall survival benefit of AT, relative to MTD, is

$$\frac{\frac{1}{\lambda_R f_{min}-\mu_{RA}}\log\left(\frac{cN_0}{R_0}\right)+\frac{1}{\lambda_R-\mu_{RM}}\log\left(\frac{M}{cN_0}\right)}{\frac{1}{\lambda_W-\mu_W-(\lambda_R f_{min}-\mu_{RM})}\log\left(\frac{R_0}{W_0}\right)+\frac{1}{\lambda_R-\mu_{RM}}\left[\log\left(\frac{M}{R_0}\right)-\frac{\lambda_R f_{min}-\mu_{RM}}{\lambda_W-\mu_W-(\lambda_R f_{min}-\mu_{RM})}\log\left(\frac{R_0}{W_0}\right)\right]}$$

$$= \frac{\frac{\lambda_R-\mu_{RM}}{\lambda_R f_{min}-\mu_{RA}}\log\left(\frac{cN_0}{R_0}\right)+\log\left(\frac{M}{cN_0}\right)}{\frac{\lambda_R(1-f_{min})}{\lambda_W-\lambda_R f_{min}+\mu_{RM}-\mu_W}\log\left(\frac{R_0}{W_0}\right)+\log\left(\frac{M}{R_0}\right)} \tag{26}$$

$$\to \frac{(\lambda_R-\mu_{RM})(\lambda_W-\lambda_R f_{min}+\mu_{RM}-\mu_W)}{(\lambda_R f_{min}-\mu_{RA})(\lambda_W-\lambda_R+\mu_{RM}-\mu_W)} \quad \text{as } \frac{N_0}{R_0}\to 0. \tag{27}$$

When measuring progression-free survival, $T_{MTD1}$ and $T_{AT1}$ are as in the case of overall survival but

$$\begin{aligned} T_{MTD2} &= \frac{1}{\lambda_R-\mu_{RM}}\left[\log\left(\frac{N_0}{R_0}\right)-\frac{\lambda_R f_{min}-\mu_{RM}}{\lambda_W-\mu_W-(\lambda_R f_{min}-\mu_{RM})}\log\left(\frac{R_0}{W_0}\right)\right], \\ T_{AT2} &= 0, \end{aligned} \tag{28}$$

and the predicted progression-free survival benefit is therefore

$$\frac{\frac{\lambda_R-\mu_{RM}}{\lambda_R f_{min}-\mu_{RA}}\log\left(\frac{cN_0}{R_0}\right)}{\frac{\lambda_R(1-f_{min})}{\lambda_W-\lambda_R f_{min}+\mu_{RM}-\mu_W}\log\left(\frac{R_0}{W_0}\right)+\log\left(\frac{N_0}{R_0}\right)} \tag{29}$$

$$\to \frac{(\lambda_R-\mu_{RM})(\lambda_W-\lambda_R f_{min}+\mu_{RM}-\mu_W)}{(\lambda_R f_{min}-\mu_{RA})(\lambda_W-\lambda_R+\mu_{RM}-\mu_W)} \quad \text{as } \frac{N_0}{R_0}\to 0. \tag{30}$$

If the treatment-induced mortality rate of resistant cells is close to zero (which is frequently a reasonable assumption) then we can simplify the limit of Equations 27 and 30 to

$$\frac{\lambda_W-\lambda_R f_{min}-\mu_W}{f_{min}(\lambda_W-\lambda_R-\mu_W)}. \tag{31}$$

If the maximum growth rates of sensitive and resistant cells are similar in the absence of treatment then we can further simplify the limit to

$$\frac{1}{f_{min}}-\frac{\lambda_W}{\mu_W}\left(\frac{1}{f_{min}}-1\right). \tag{32}$$

This last approximation makes clear that the predicted maximum benefit of AT, relative to MTD, strongly depends on the relative fitness of resistant cells when rare, $f_{min}$.

When comparing our analytical approximations with results of numerical simulations, we need to account for growth of the sensitive population before the first treatment bolus. This means replacing $W_0$ and $N_0$ in Equations 21, 22, 24, 26 and 29 by (respectively)

$$W_0'=\frac{(1+e^{\theta\lambda_W})W_0}{2}, \quad N_0'=\frac{(1+e^{\theta\lambda_W})N_0}{2}. \tag{33}$$

Finally, we note that by rearranging the expression for $T_{AT1}$ in Equation 25, we can derive an expression for calculating $f_{min}$ from more readily measurable parameter values:

$$f_{min}=\frac{1}{\lambda_R}\left(\frac{1}{T_{AT1}}\log\frac{N_0}{2R_0}+\mu_{RA}\right), \tag{34}$$

where we assume $c=\frac{1}{2}$ (since the result is not sensitive to this value).

# Spatial computational model

## Program structure

The program [1] was written in the C language with structure as outlined in Algorithm 1.

```
Create initial tumour;
t = 0;
while t < time_limit and no tumour cell has reached edge of grid do
    Calculate consumption rates (Equations 36 and 40);
    Solve diffusion equations (Equation 35);
    for each cell i do
        Calculate proliferation rate P_i and death rate M_i (Equations 38 and 39)
    end
    Record state at time t;
    for each cell i do
        if oxygen < survival_threshold then add cell to kill_list;
    end
    events_count = 0;
    while events_count < update_limit do
        Kill the next cell in kill_list;
        Increment events_count;
    end
    while events_count < update_limit do
        Calculate time until next event: δt ~ Exp(1/∑_i(P_i + M_i));
        Randomly select cell k, with Prob(k = K) = (P_K + M_K)/∑_i(P_i + M_i);
        Randomly select cell proliferation or death, with weights P_k and M_k;
        if proliferation then
            if cell k has space to proliferate then
                Add new cell;
                Increment events_count;
                Update proliferation statuses of nearest neighbours;
            end
        end
        else
            Remove cell k;
            Increment events_count;
            Update proliferation statuses of nearest neighbours;
        end
        Increase time t by δt;
    end
end
Record final state;
```

**Algorithm 1:** Outline description of the algorithm used to implement the model.

## Solving diffusion equations

Oxygen and CDK inhibitor diffuse from the surrounding medium into the tumour spheroid and are consumed by cells. As is conventional, we assume that diffusion occurs much faster than cell division or death, and so chemical concentrations are always at equilibrium. The equation to be solved for each location $(x, y)$ on the grid is therefore [2]

$$D\nabla^2 c(x, y) - f(c, x, y) = 0, \tag{35}$$

where $D$ is the diffusion coefficient, $\nabla^2 c$ is the local concentration of the chemical (either oxygen or CDK inhibitor), and $f$ is the chemical consumption function. The equations are solved numerically in the smallest rectangle containing the tumour by the method of successive overrelaxation with Chebyshev acceleration [2]. The solution depends on the cell diameter, which is assumed to be 17 μm (consistent with cell density of $2 \times 10^8$ cells cm$^{-3}$) [3, 4].

## Oxygen diffusion and consumption, and necrosis

We include an oxygen field in our model to recreate the necrotic core observed in tumour spheroids. Our results are insensitive to how this necrotic core arises, and we therefore make parsimonious assumptions regarding oxygen diffusion and consumption based on the experimental literature.

Consistent with previous studies [4, 5, 6], we assume that the medium surrounding the tumour spheroid has a uniform oxygen concentration of $2.2 \times 10^{-7}$ mol cm$^{-3}$ (equivalent to 0.22 mM, or a partial pressure of approximately 100 mmHg), and the oxygen diffusion rate is $2 \times 10^{-5}$ cm$^2$ s$^{-1}$.

In monolayer culture, oxygen consumption is approximately constant at relatively high oxygen concentrations, with a steep decline only at very low oxygen levels [7]. Consistent with previous models of tumour growth [4, 5], we assume the following relationship, which was derived experimentally using the EMT6/Ro mouse mammary tumour cell line [8]:

$$Q = Q_{max} \frac{48g}{48g + 1}, \tag{36}$$

where $Q$ is oxygen consumption, $g$ is oxygen concentration relative to $2.2 \times 10^{-7}$ mol cm$^{-3}$, and $Q_{max}$ is the maximum possible oxygen consumption rate.

Whereas the value of $Q_{max}$ has been measured as $1.0 \times 10^{-8}$ mol cm$^{-3}$ s$^{-1}$ [3], this rate does not account for quiescence, which is likely to reduce oxygen consumption inside tumours and tumour spheroids. Indeed, several previous computational models have assumed that quiescent cells consume less oxygen than proliferating cells (e.g. [9] assumes half as much; [10] assumes one fifth). The problem then is to define robust, realistic criteria for quiescence. For simplicity, because it has been shown to make very

little difference to model outcomes [7], we instead assume that all cells consume oxygen at a maximum rate of $5 \times 10^{-9}$ mol cm$^{-3}$ s$^{-1}$.

We further assume that cells with relative oxygen concentration $g < 0.001$ die instantly, which results in a viable rim approximately 8 cells wide.

## Oxygen effects on proliferation

In monolayer culture, cancer cell proliferation rates, like oxygen consumption rates, are nearly constant at high oxygen concentrations and decline steeply at low oxygen levels. While more sophisticated models have been proposed (e.g. [11]), the relationship can be approximated by Michaelis-Menten kinetics. Consistent with previous experimental and modelling studies [8, 12], we assume that the proliferation rate $P$ of drug-sensitive cells in the absence of CDK inhibitor is

$$P = P_{max} \frac{30g}{30g + 1}, \tag{37}$$

where $P_{max}$ is the maximum possible proliferation rate. To account for a 10% reduction in maximum growth rate due to the fitness cost of resistance, and to rescale such that the maximum proliferation rate is 1, we set $P_{max} = 31/30$ for drug-sensitive cells and $P_{max} = 0.9 \times 31/30$ for drug-resistant cells. Model outcomes were relatively insensitive to varying the fitness cost of resistance.

## Crowding effects on proliferation

Equation 37 does not realistically account for extensive quiescence observed in tumour spheroids, which is thought to result from cell contact effects or an unknown growth inhibitory factor [8]. We therefore assume that a cell will fail to divide if it has insufficient space to do so. Specifically, cells can proliferate only if there is at least one unoccupied site within a disc-shaped neighbourhood of radius $d$ cell widths. Sites are unoccupied if they do not contain a living cell (i.e. they contain either medium or a dead cell). First the algorithm checks, in random order, the sites within 1.5 cell diameters of the centre of the focus cell. If a site is only partly within the disc then it is checked with probability proportional to its overlap with the disc (when determining the overlap, cells are also treated as discs, not squares). If no unoccupied site is found then the search is extended to a disc of radius 2.5 cell diameters. The process continues up until disc radius $d$ (Supplementary Fig. 7A). If an unoccupied site is found adjacent to the focus cell then a daughter cell is placed at that site. If an unoccupied site is found further away then the cells lying on a straight line between the focus cell and the unoccupied site are shifted one space along that line to create an unoccupied site next to the focus cell (Supplementary Fig. 7B). This budging method thus enables proliferation of cells located close to the periphery as well as those on the periphery. We set $d = 2.5$ so that

typically only the two or three outermost layers of cells were able to proliferate, as has been observed in tumour spheroids [13]. We also tested the model with $d = 20$, which (because the viable rim is less than 20 cells wide) effectively removes crowding inhibition of proliferation.

## CDK inhibitor effects on proliferation and cell death

The effects of oxygen, glucose and pH have been found to combine multiplicatively to determine cell proliferation rate [8]. We assume that effects of oxygen (Equation 37) and the CDK inhibitor also combine multiplicatively.

In monolayer culture experiments, the doubling times of sensitive cells, relative to the doubling times in the absence of CDK inhibitor NU6102, were 0.76 at 5 μM NU6102, 0.55 at 10 μM, and 0.03 at 20 μM, indicating an approximately linear relationship between proliferation rate and CDK inhibitor dose. Population growth at 20 μM was even lower in tumour spheroid experiments than in monolayer culture. Accordingly, we assume that the relative proliferation rate $P$ for sensitive cells decreases linearly as the CDK inhibitor dose $h$ increases, such that $P = 1$ when $h = 0$ and $P = 0.002$ when $h = 20\mu$M. Hence

$$P = P_{max}\left(\frac{30g}{30g+1}\right)\max\left(1 - 0.0499h, 0\right). \tag{38}$$

For sensitive cell death rate $M$ we likewise assume a linear dependence on CDK inhibitor dose, such that $M = 0.55$ when $h = 50\mu$M (as in our monolayer culture experiments) and $M = 0.003$ (slightly higher than $P$) when $h = 20\mu$M. Hence

$$M = \max\left(0.0182h - 0.361, 0\right). \tag{39}$$

We assume that resistant cells are unaffected by the CDK inhibitor, so that the proliferation and death rates of cells at maximum oxygen concentration (such as at the tumour spheroid periphery) are as in Supplementary Fig. 7C.

To facilitate comparison with our experimental results, we rescaled the relative time units of the model into days, such that the model tumour spheroid growth curves in the absence of treatment approximately coincided with those of the experiments. With proliferation threshold $d = 2.5$, one day then corresponds to 0.6 doubling times of a sensitive cell in optimal conditions (i.e. located on the tumour spheroid periphery in the absence of treatment).

## CDK inhibitor diffusion and consumption rates

We assume that the cell consumption rate of CDK inhibitor is small relative to its diffusion rate, which ensures that the CDK inhibitor concentration at the centre of the spheroid is not much less than the concentration in the medium. If the consumption rate were

higher then, at the 20 μM dose, cells exactly on the periphery would encounter a 20 μM CDK inhibitor concentration but cells one or two cell diameters away from the periphery would receive a substantially lower concentration. Given our assumed proliferation and death rates (Supplementary Fig. 7C), the spheroid would then grow due to proliferation of these internal cells, even if all cells were sensitive to the inhibitor, which is inconsistent with our experimental results (in which spheroid volume was approximately static at 20 μM dose). Therefore we must assume that the gradient of inhibitor concentration across the spheroid is relatively shallow, which requires the consumption rate of CDK inhibitor to be small relative to its diffusion rate.

Specifically, we assume a diffusion rate of $5 \times 10^{-6}$ cm$^2$ s$^{-1}$ (consistent with the large molecular weight of the drug) and we set

$$R = R_{max} \frac{48g}{48g + 1}, \tag{40}$$

where $R$ is the consumption rate, and $R_{max} = 1.0 \times 10^{-11}$ mol cm$^{-3}$ s$^{-1}$ is the maximum possible consumption rate. The parameter values in this equation are necessarily somewhat arbitrary as the pharmacokinetics of NU6102 in tumour spheroids are unknown, but the model's qualitative behaviour and quantitative output are insensitive to these parameters, provided that the inhibitor gradient across the tumour spheroid is relatively shallow.

## Initial state

We compared model outcomes for two different initial states. In the first scenario, we initiated the model with cells arranged in a disc of radius 7 cells, at the centre of the grid. The initial population was therefore 156 cells (approximately $\pi \times 7^2$), corresponding to a cross section of a three-dimensional tumour spheroid comprising approximately 1,500 cells ($\frac{4}{3}\pi \times 7.1^3$), which was the size of the initial population in the *in vitro* experiments. In the experiments, CDK inhibitor was added after 4 days of growth, when the tumour spheroid volume reached approximately 0.03 mm$^3$. Accordingly, before adding the CDK inhibitor in the model, we ran the simulation for an initial growth phase, which was long enough for the tumour spheroid volume to reach approximately 0.03 mm$^3$. Therefore in this case, the spatial distribution of resistant cells was uniformly random at the start of the simulation, but was subsequently influenced by competition with sensitive cells before the start of treatment.

For the alternative initial state, we created the tumour spheroid with a volume of approximately 0.03 mm$^3$ and began treatment immediately, so that resistant cells were uniformly randomly distributed at the start of treatment.

## Radius measurement

To find the relationship between cell number and tumour spheroid radius in the model, we measured the radius in the absence of treatment as the distance from the centre of the grid to the outermost live cell, averaged over eight directions at 45 degree intervals, and over many replicates. We then used linear regression to characterise this relationship before the emergence of a necrotic core (when radius is proportional to the square root of population size) and after the emergence of a necrotic core (when radius grows linearly with population size). Thus we obtained a reference curve for the relationship between population size and radius in approximately circular tumour spheroids. Since CDK-treated tumour spheroids in the model can be less regularly shaped than *in vitro*, we used the reference curve to convert from population size to a notional radius that facilitates comparison with experimental results. In converting the units of the radius we assumed a cell density of $2 \times 10^8$ cells cm$^{-3}$ [3, 4].

# Supplementary references


[1] Robert Noble. HyCAT version 0.0.1, 2017. URL `https://github.com/robjohnnoble/HyCAT`. doi: 10.5281/zenodo.838190.

[2] William T Vetterling, Saul A Teukolsky, Brian P Flannery, and William H Press. *Numerical Recipes in C The Art of Scientific Computing*. Cambridge University Press, 2nd edition, 2002. ISBN 0521431085.

[3] BA Wagner, S Venkataraman, and GR Buettner. The rate of oxygen utilization by cells. *Free Radical Biology and Medicine*, 51(3):700–712, 2011.

[4] JJ Casciari, SV Sotirchos, and RM Sutherland. Mathematical modelling of microenvironment and growth in EMT6 / Ro multicellular tumour spheroids. *Cell proliferation*, 25(1):1–22, 1992.

[5] Yangjin Kim, Magdalena A Stolarska, and Hans G Othmer. A Hybrid Model For Tumor Spheroid Growth In Vitro I: Theoretical Development And Early Results. *Mathematical Models and Methods in Applied Sciences*, 17:1773–1798, 2007.

[6] David Robert Grimes, Catherine Kelly, Katarzyna Bloch, and Mike Partridge. A method for estimating the oxygen consumption rate in multicellular tumour spheroids. *Journal of the Royal Society Interface*, 11:20131124, 2014.

[7] David Robert Grimes, Alexander G Fletcher, and Mike Partridge. Oxygen consumption dynamics in steady-state tumour models. *Royal Society Open Science*, 1: 140080, 2014.

[8] Joseph J Casciari, Stratis V Sotirchos, and Sutherland Robert M. Variations in Tumor Cell Growth Rates and Metabolism With Oxygen Concentration, Glucose Concentration, and Extracellular pH. *Journal of cellular physiology*, 151:386–394, 1992.

[9] Yi Jiang, Jelena Pjesivac-Grbovic, Charles Cantrell, and James P Freyer. A multi-scale model for avascular tumor growth. *Biophysical journal*, 89(6):3884–94, Dec 2005. ISSN 0006-3495.

[10] Philip Gerlee and Alexander R A Anderson. An Evolutionary Hybrid Cellular Automaton Model of Solid Tumour Growth. *Journal of theoretical biology*, 246(4): 583–603, 2007.

[11] T Alarcón, H M Byrne, and P K Maini. A mathematical model of the effects of hypoxia on the cell-cycle of normal and cancer cells. *Journal of theoretical biology*, 229(3):395–411, Aug 2004.

[12] JP Ward and JR King. Mathematical modelling of avascular-tumour growth. *Mathematical Medicine and Biology*, 14:39–69, 1997.

[13] R M Sutherland. Cell and environment interactions in tumor microregions: the multicell spheroid model. *Science (New York, N.Y.)*, 240(4849):177–84, Apr 1988.
