# Supplementary material for: Spatial competition constrains resistance to targeted cancer therapy
Source: Nat Commun. 2017 Dec 8;8:1995. doi: 10.1038/s41467-017-01516-1 (PMC5722825; doi:10.1038/s41467-017-01516-1)
Supplement: Supplementary file 3 — Description of Additional Supplementary Files [file 41467_2017_1516_MOESM3_ESM.pdf]

Description of Additional Supplementary Files

File Name: Supplementary Movie 1
Description: Simulation of tumour spheroid growth with initial 1% frequency of resistant cells, grown without the inhibitor.

File Name: Supplementary Movie 2
Description: Simulation of tumour spheroid growth with initial 1% frequency of resistant cells, grown at 5μM NU6102.

File Name: Supplementary Movie 3
Description: Simulation of tumour spheroid growth with initial 1% frequency of resistant cells, grown at 10μM NU6102.

File Name: Supplementary Movie 4
Description: Simulation of tumour spheroid growth with initial 1% frequency of resistant cells, grown at 20μM NU6102.

File Name: Supplementary Movie 5
Description: Simulation of tumour spheroid growth with initial 1% frequency of resistant cells, grown at 50μM NU6102.

File Name: Supplementary Data 1
Description: Results of microarray analysis of gene expression in WT, WT cells treated with 20μM NU6102 for 24h, R50 and $CDK^{-/-}$ cells.
