# Supplementary figures and images for: Spatial competition constrains resistance to targeted cancer therapy
Source: Nat Commun. 2017 Dec 8;8:1995. doi: 10.1038/s41467-017-01516-1 (PMC5722825; doi:10.1038/s41467-017-01516-1)

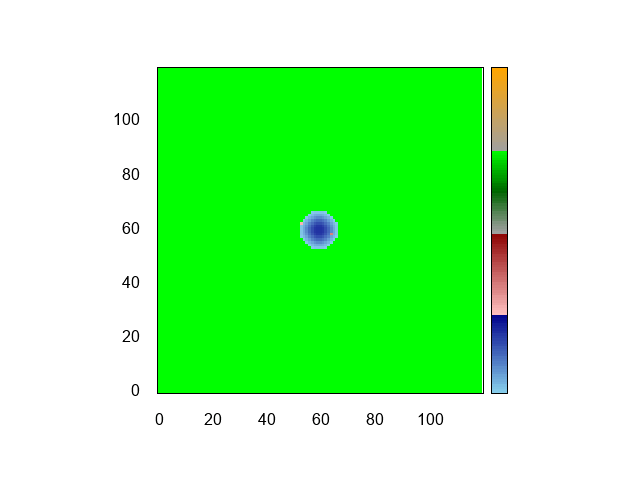

Supplement: Supplementary file 4 — Supplementary Movie 1 [file 41467_2017_1516_MOESM4_ESM.gif]

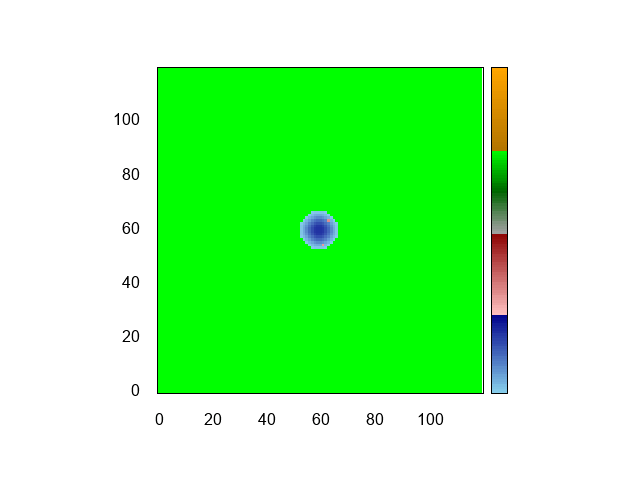

Supplement: Supplementary file 5 — Supplementary Movie 2 [file 41467_2017_1516_MOESM5_ESM.gif]

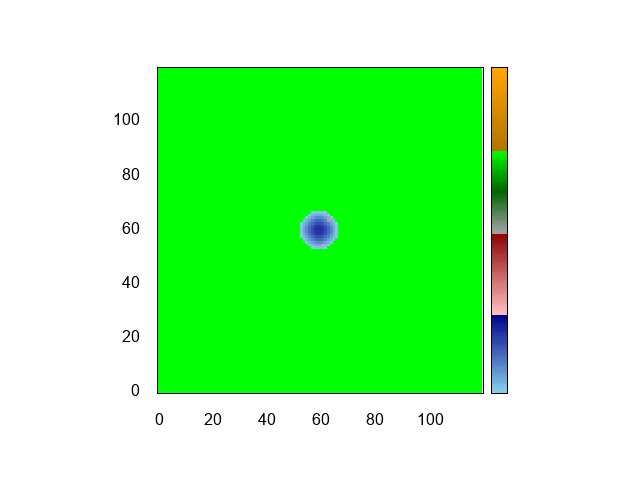

Supplement: Supplementary file 6 — Supplementary Movie 3 [file 41467_2017_1516_MOESM6_ESM.gif]

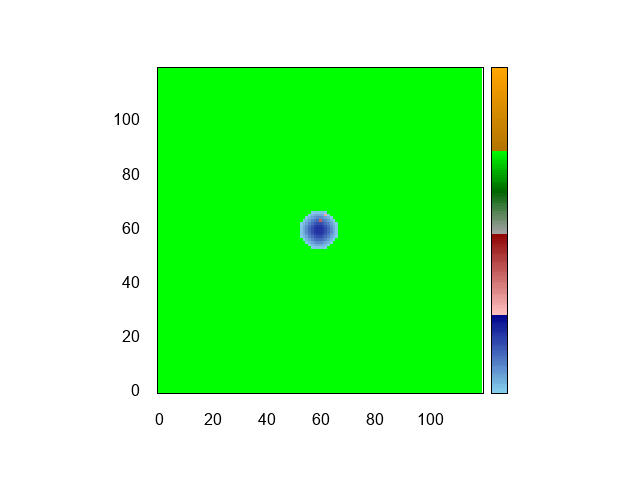

Supplement: Supplementary file 7 — Supplementary Movie 4 [file 41467_2017_1516_MOESM7_ESM.gif]

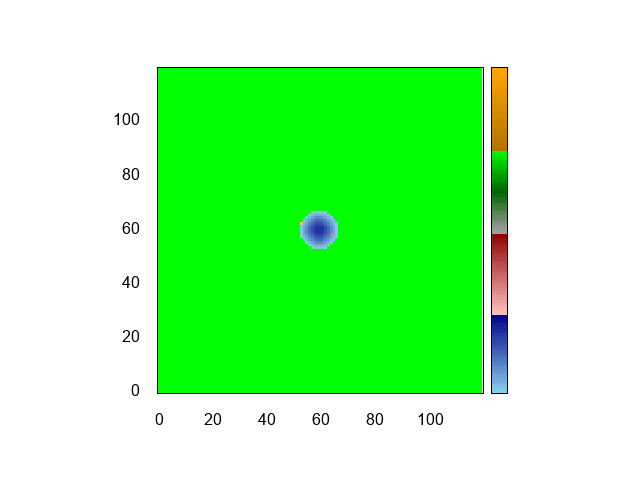

Supplement: Supplementary file 8 — Supplementary Movie 5 [file 41467_2017_1516_MOESM8_ESM.gif]
